# Supplementary material for: Exploring the Hydrogen-Induced Amorphization and Hydrogen Storage Reversibility of Y(Sc)0.95Ni2 Laves Phase Compounds
Source: Materials (Basel). 2021 Jan 7;14(2):276. doi: 10.3390/ma14020276 (PMC7827125; doi:10.3390/ma14020276)
Supplement: Supplementary file 1 [file materials-14-00276-s001.pdf]

materials

Supplementary

Exploring the Hydrogen-Induced Amorphization and Hydrogen Storage Reversibility of $Y(Sc)_{0.95}Ni_2$ Laves Phase Compounds

Shiqian Zhao, Hui Wang * and Jiangwen Liu

School of Materials Science and Engineering and Guangdong Provincial Key Laboratory of Advanced Energy Storage Materials, South China University of Technology, Guangzhou 510641, China; 201710103269@mail.scut.edu.cn (S.Z.); mejwliu@scut.edu.cn (J.L.)

* Correspondence: mehwang@scut.edu.cn; Tel.: +86-(020) 87112762

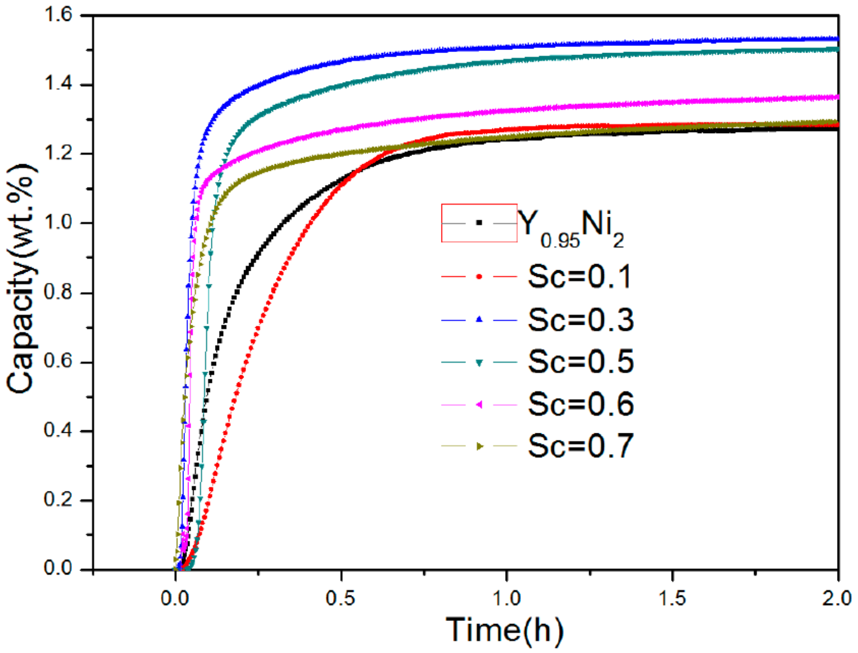

Figure S1. Hydriding kinetic curves of Y-Sc-Ni compounds with different Sc contentsat 40 °C.
